# Phytochemical and acute toxicity of ethanolic extract of *Enantia chlorantha* (oliv) stem bark in albino rats

**DOI:** 10.2478/intox-2013-0023

**Published:** 2013-09

**Authors:** Olamide E. Adebiyi, Mathew O. Abatan

**Affiliations:** Department of Veterinary Physiology, Biochemistry and Pharmacology, University of Ibadan, Ibadan, Nigeria

**Keywords:** *Enantia chlorantha*, acute toxicity, serum biochemistry, rats

## Abstract

It is presumed that drugs sourced from herbs have lesser side effects than allopathic drugs. *Enantia chlorantha* is widely used in herbal medicine for the treatment of several ailments such as jaundice, malaria, fever, infective hepatitis, *etc.* However its toxicity profiles are not well documented. The effects of ethanolic extract of *E. chlorantha* stem bark on body weight changes, biochemical and haematological parameters as well as histology of vital organs (heart, kidneys and liver) were assessed. Also, the phytochemical constituent of the plant was analysed. Albino rats of both sexes were randomly divided into five groups (A–E) of five rats each and the ethanolic extract of *E. chlorantha* stem bark extract was administered by oral gavage in a single dose. Group A rats were administered 500 mg/kg of the extract, group B; 1000 mg/kg, group C; 2000 mg/kg, group D; 3000 mg/kg and group E rats received distilled water (10 ml/kg) and served as control. The extract caused significant (*p<*0.05) decreases in the levels of packed cell volume, haemoglobin concentration and red blood cell counts in a dose dependent manner. Further, significant alterations were not observed in the serum biochemical parameters analysed (AST, ALP, ALT, blood urea nitrogen, total protein, albumin, globulin and bilirubin). In addition, the extract at 1000, 2000 and 3000 mg/kg caused congestion in the heart and kidney of experimental rats. These results suggest that oral administration of *E. chlorantha* may produce severe toxic effects at relatively high doses, thus caution should be exercised in its use.

## Introduction

Natural products have been, and have remained, the cornerstone of health care. Present estimates show that 80% of the world's population still rely on traditional medicine for their health care needs (Farnsworth *et al.*, 1985).

*Enantia chlorantha* is widely distributed along the coasts of West and Central Africa. It is also very common in the forest regions of Nigeria. It is an ornamental tree which may grow up to 30 m high, with dense foliage and spreading crown. The outer bark which is thin and dark brown is fissured geometrically while the inner bark is brown above and pale cream beneath. The stem is fluted and aromatic while the elliptic leaves are about 0.14–0.15 m long and 0.05–0.14 m broad (Iwu, 1993).

Studies have reported the possible use of the plant in conditions such as rickettsia fever, cough and wounds, typhoid fever and infective hepatitis or jaundice (Gill, 1992). It has also been revealed that the plant possesses antipyretic (Agbaje & Onabanjo, 1998) as well as antimicrobial and antimalarial activities (Adesokan *et* *al.,* 2007; Odugbemi *et al.,* 2007; Fasola *et al.,* 2011). In Cameroon, stem bark extract of *E. chlorantha* is used to treat jaundice and urinary tract infections (Adjanohoun *et al.*, 1996).

The medicinal properties of the plant may be due to one or more of its phytochemical constituents. However, some of these compounds may be toxic, and thus the plants containing them could confer varied levels of toxicity to an individual consuming them. A plant, *Hilleria latifolia*, which is used as analgesic and the treatment of asthma, has been reported to induce cholinergic signs like defecation, salivation and urination in experimental animals (Wonder *et al.,* 2011). Some plants may therefore be inherently dangerous, containing naturally occurring toxins, often with cytotoxic, cardiotoxic effects, or some other toxic properties (Humphrey & McKenna, 1997). *E. chlorantha* is not an exception. Unfortunately, most of the users of this plant do not have the knowledge of its adverse effects, toxicity, and neither of its other beneficial properties (Agbaje & Babatunde, 2005). Therefore, in order to have a standard natural plant product, preliminary studies have to be done to evaluate possible risks such as, undesirable effects, overdose or poisoning. Our study was conducted to identify the major chemical groups contained in the ethanolic stem bark extract of *E. chlorantha*, its hematologic and serum biochemistry variables, as well as histopathological changes following a single oral intake in albino rats. This should provide preliminary safety information regarding *E. chlorantha* as well as establish the toxicological limits in rats.

## Material and methods

### Experimental animals

Albino rats of both sexes weighing between 150 and 190 g were randomly divided into five groups (A–E) of five rats each. They were housed at the Animal House of the Faculty of Veterinary Medicine, University of Ibadan. They were kept in rat cages and fed rat pellets (Animalcare^®^ Feeds Ltd., Ogere, Nigeria) and allowed free access to clean fresh water in bottles *ad libitum.* The study was approved by the Animal Ethics Committee of the Faculty of Veterinary Medicine, University of Ibadan.

### Plant material and authentication

The plant samples were collected from the local region between September and October, 2012 (rainy season). The plant was identified and authenticated at the Forestry Research Institute of Nigeria, Ibadan and voucher specimen (FHI. 109950) was preserved at the herbarium.

### Preparation of the plant material

The stem bark was cleaned to remove adhering dirt, air-dried for two weeks and was ground into powder using an electric blender (Blender/Miller III, model MS-223, Taiwan, China). Extraction was carried out by cold maceration of 800 g of the coarse powder with 5L of 70% ethanol for 72 h, with constant shaking using the GFL shaker (no. 3017GBh, Germany). The resultant mixture was filtered using Whatman filter paper (No.1) and the filtrate was concentrated to dryness *in vacuo* at 40 °C using rotary evaporator to give a yield of 12% w/w of the extract. Aliquot portions of the extract were weighed and dissolved in distilled water for use in the study.

### Preliminary phytochemical analysis

Standard methods were used to detect the nature of phytoconstituents present in the ethanolic extract of *E. chlorantha* stem bark (Kokate, 1998; Khandelwal, 2005).

### Acute toxicity study

The test was carried out following the methods described by the Organisation for Economic Cooperation and Development (OECD, 2001); rats were deprived of food for 24 hours prior to extract administration and randomly divided into five groups A–E. Groups A, B, C and D were administered 500, 1000, 2000 and 3000 mg/kg body weight single dose of ethanolic extract of *Enantia chlorantha* stem bark, respectively, by oral gavage, group E received distilled water (10 ml/kg) only and was used as control. Thereafter, food intake was resumed. All the animals were acclimatized to laboratory conditions for two weeks before commencement of experiment.

### Clinical observations

The observation period was 14 days post administration. The monitoring of the parameters commenced immediately after administrating the extract. The rats were kept under the same conditions and observed at 0 hr, 1 hr, 2 hrs, 4 hrs, 6 hrs, 8 hrs, 24 hrs, and daily thereafter, for a total of 14 days, for signs of toxicity, which included but were not limited to paw-licking, motor activity, tremors, convulsions, posture, spasticity, opisthotonicity, ataxia, sensations, pilo-erection, ptosis, lacrimation, exopthalmos, salivation, diarrhoea, writhing, skin colour, respiratory rate and mortality (Jaykaran *et al.,* 2009; Salawu *et al.,* 2009).

### Body weight

Body weights of animals were measured shortly before the test substance was administered and weekly thereafter.

### Blood collection

Fifteen days after the oral administration of the extract, blood was drawn from each of the animal by cardiac puncture for haematology and serum biochemistry.

### Haematology

Haematological parameters including Packed cell volume (PCV), haemoglobin (Hb), red blood cells (RBC), white blood cells (WBC), platelets, mean corpuscular volume (MCV), mean corpuscular haemoglobin (MCH), and mean corpuscular haemoglobin concentration (MCHC) were determined by an automatic analyzer (BC-3000 Plus Auto Hematology Analyzer, Shenzhen Mindray Bio-Medical Electronics Co. Ltd, China). Total leukocyte and leukocyte differential counts were also determined.

### Serum biochemistry

The blood collected was allowed to clot and was centrifuged at a speed of 3500 rpm for 10 minutes to obtain serum. The serum was stored at –20 °C until tested. Biochemical analyses were performed on serum collected for the determination of the following parameters: aspartate transaminase (AST), alanine transaminase (ALT), alkaline phosphatase (ALP), total bilirubin, total protein, albumin, blood urea nitrogen (BUN). Globulin was obtained from the difference between total protein and albumin. All analyses were carried out using Flexor Junior Automated Clinical Chemistry Analyser (Vital Scientific, AC Dieren, The Netherlands).

### Histopathological examination

The animals were sacrificed after blood collection; small pieces of vital organs (heart, kidneys and liver) were collected in 10% formaldehyde solution for histopathological study. The organ tissues were processed and embedded in paraffin wax and sections were made of about 4–6 µm in thickness. After staining with haematoxylin and eosin (H&E), slides were examined under microscope (Olympus, Japan) for histopathological changes and photographed.

### Statistical analysis

All data were expressed as mean ± standard error of mean (SEM), comparison was performed by the student t-test using Graphpad Prism version 4.00 for Windows, Graphpad Software. A probability value of *p≤*0.05 was considered statistically significant.

## Results

Phytochemical screening of the ethanolic extract of *E. chlorantha* stem bark revealed the presence of alkaloid and reducing sugar (Table 1). No mortality was observed in the experimental animals during this study. Furthermore, no significant change in body weight was observed in the animals in the test and control groups.

**Table 1:** Qualitative phytochemical evaluation of the ethanolic extract of *Enantia chlorantha* (Oliv) Stem Bark.

Constituents	Observation
Alkaloid	+
Glycosides	–
Flavonoid	–
Saponins	+
Reducing Sugar	+
Tannins	–
Phlobatanins	–
Steroids	–
Cardiac glycoside	–
Phenol	–
Resin	–

+ positive; – not detected

**Table 2:** Effect of graded doses of ethanol extract of *Enanthia chlorantha* stem bark on body weights of rats.

	Group A	Group B	Group C	Group D	Group E
Day 1	176.02±2.45	179.11±1.67	182.50±1.76	184.60±2.05	182.00±1.83
Day 8	175.00±1.23	177.09±1.58	177.25±1.08	180.05±1.35	182.50±0.23
Day 15	174.63±0.67	176.21±2.11	179.05±1.30	178.00±1.20	184.75±0.94

PCV was reduced in all the test groups when compared with that of the control. These decreases were statistically significant (*p<*0.05) in groups B–D when compared with that of the control (Table 3); the decrease was 18.9%, 30.3% and 25% of the control in groups B, C and D. Furthermore, a statistically significant (*p<*0.05) reduction was observed in Hb count of the groups administered 2000 mg/kg and 3000 mg/kg of the ethanolic extract of *E. chlorantha* stem bark when as with the control group. A significant (*p<*0.05) increase in white blood cell (WBC) count was also observed in the 2000 mg/kg and 3000 mg/kg groups; the increase was 55.7% and 51.1%, respectively, relative to the control and mean corpuscular volume (MCV) was also significantly decreased in the 2000 mg/kg and 3000 mg/kg groups; the decrease was 22.9% and 19.5%, respectively, relative to the control. There was no significant difference in the percentage granulocytes, lymphocytes and monocytes count in all the test groups when compared with the control group. There was also a statistically significant decrease in platelet counts in the 1000, 2000 and 3000 mg/kg groups (Table 3), this decrease was 31.2%, 53.5% and 48.3%, respectively, relative to the control.

**Table 3:** Effect of the graded dose of ethanol extract of *Enanthia chlorantha* stem bark on haematology of albino rats on day 15 after the administration of *the* extract on day 1.

	Group A	Group B	Group C	Group D	Group E
PCV (%)	38.59±0.13^a^	35.67±2.84^b^	30.67±4.10^b^	31.67±4.67^b^	44.00±0.58^a^
Hb (g/dl)	12.83±0.01^a^	11.83±0.97^a^	10.50±1.60^b^	10.20±1.32^b^	13.66±0.20^a^
RBC × 10^6^/cm^3^	8.30±1.19	8.35±1.49	8.35±1.89	8.32±1.54	9.24±1.43
WBC x10^3^/cm^3^	6.29±1.13^a^	7.97±1.29^a^	9.87±1.27^b^	9.60±0.12^b^	6.34±3.23^a^
% Lymphocyte	60.3±0.13	64.00±0.10	61.67±0.33	60.00±0.10	60.40±2.86
% Monocytes	19.06±0.46	16.23±0.62	18.05±0.28	18.27±1.04	18.04±0.89
% Granulocyte	20.00±0.33	19.67±0.33	20.00±0.58	21.00±0.58	21.00±1.97
Platelets	10.00±0.01^a^	8.87±1.33^b^	6.00±2.31^b^	6.67±2.67^b^	12.89±1.74^a^
MCV (fl)	46.81±0.46^a^	42.67±1.55^a^	36.73±3.36^b^	38.36±3.18^b^	47.63±1.11^a^
MCH (pg)	12.89±0.01	14.67±1.76	13.00±2.52	11.33±0.88^a^	12.09±1.03
MCHC	33.25±0.02	33.17±0.10	34.23±1.03	32.34±0.10	32.10±0.36

n=5; Mean ± S.E.M (Standard Error of Mean); Means with different superscripts within rows are significantly different at *p<*0.05; PCV = packed cell volume; Hb = haemoglobin count; RBC = red blood cell count; WBC = White blood cell count; MCV = mean corpuscular volume; MCH = mean corpuscular haemoglobin; MCHC = mean corpuscular haemoglobin concentration

Slight reduction was observed in the levels of total serum protein, albumin and globulin in the test groups when compared with the control group; however these decreases were not statistically significant. No significant alterations were observed in the analysed liver enzymes (AST, ALT and ALP), blood urea nitrogen (BUN), and serum bilirubin levels in any of the groups when compared with the control group (Table 4).

**Table 4:** Effect of graded doses of ethanol extract of *Enanthia chlorantha* on serum biochemistry of albino rats on day 15 after the administration of *the* extract on day 1.

	Group A	Group B	Group C	Group D	Group E
TP (g/dl)	2.65±0.22	2.12±0.59	3.17±0.17	2.70±0.65	3.10±0.67
ALB (g/dl)	0.95±0.05	1.03±0.12	1.03±0.03	0.90±0.21	1.02±0.04
GLB (g/dl)	1.90±0.17	1.60±0.32	2.13±0.13	1.80±0.45	2.12±0.59
AST (U/L)	36.50±0.63	39.00±3.79	41.67±3.93	36.67±6.67	38.8±1.88
ALT (U/L)	37.25±1.03	35.33±3.71	39.00±4.58	39.67±6.22	36.6±1.50
ALP (U/L)	44.00±2.94	41.00±3.22	45.33±3.71	48.67±4.81	40.6±3.31
BUN (mg/dl)	5.73±0.28	6.02±3.63	4.91±1.06	5.33±0.75	5.72±0.75
BIL (mg/dl)	1.32±0.36	1.73±0.32	1.60±0.31	1.40±0.40	1.71±0.32

n=5; Mean ± S.E.M (Standard Error of Mean); Means with different superscripts within rows are significantly different at *p<*0.05; TP = Total Protein; ALB = Albumin; GLB = globulin; AST = Aspartate aminotransferase; ALT = Alanine aminotransferase; ALP = Alkaline phosphatase; BIL = Billirubin; BUN = Blood urea nitrogen

**Figure 1:**
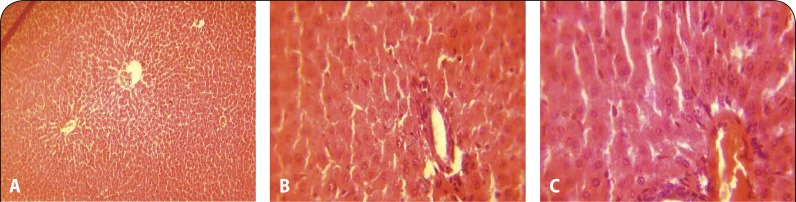
**A:** Liver of control groups showing no visible histopathological lesion (H&E stain ×40). **B:** Liver of group administered with 1000 mg/kg showing diffused proliferation of Kupffer cells between the hepatocytes and inflammatory cells infiltration (H&E stain ×100). **C:** The liver section of rats administered with extract of *E. chlorantha* at 3000 mg/kg showing periportal cellular infiltration by mononuclear cells (H&E stain ×100).

**Figure 2:**
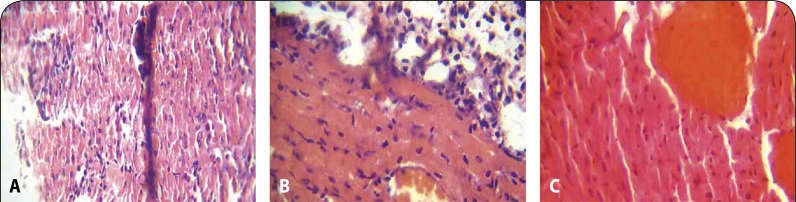
**A:** Heart of control groups showing nobvisible histopathological lesion (H&E stain ×100). **B:** Heart of test groups administered with 2000 mg/kg of the stem bark extract of *E. chlorantha* showing mild to moderate congestion of coronary vessels (H&E stain ×100). **C:** Moderate to marked congestion of coronary vessels at 3000 mg/kg (H&E ×100).

**Figure 3:**
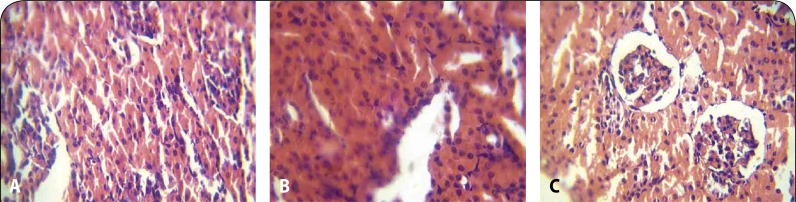
**A:** Kidney of control group showing no visible histopathological lesion in the kidney (H&E ×100). **B:** Mild to moderate renal vein congestion (H&E ×100) at 2000 mg/kg. **C:** Renal parenchyma shows tubular necrosis and hyaline degenerative changes at 3000 mg/kg (H&E ×100).

Histopathological examination revealed moderate to marked coronary and cortical congestion in the heart and kidney, respectively, of rats administered 2000 and 3000 mg/kg of the extract (Figures 2B, 2C, 3B and 3C) compared to no visible lesion in the control group. Mild periportal cellular infiltration by mononuclear cells was also observed in the liver section of rats administered the solvent extract at 1000, 2000 and 3000 mg/kg (Figure 1B).

## Discussion

The safe use of the extracts tested and of active substances they contain, explain probably their common uses by traditional healers in the treatment of numerous human and animal diseases (DMP, 2004; CAPES, 2006,). Alkaloids, saponins and reducing sugar were observed in the stem bark extracts of *E. chlorantha*. The therapeutic properties of these large chemical groups have been reported by various authors (Delaveau, 1988; Cowan, 1999).

Treatment with ethanolic extracts of *E. chlorantha* stem bark (500 mg/kg, 1000 mg/kg, 2000 mg/kg and 3000 mg/kg) was tolerated by all the animals, the signs noticed within 24 hours included loss of appetite, reduced mobility, dullness and general weakness; these signs were not seen in the 500 mg/kg dose group but progressed and became increasingly pronounced as the dose increased towards 3000 mg/kg. However no death was recorded, indicating the LD_50_ of *E. chlorantha* stem bark is approximately higher than 3000 mg/kg, and thus it is relatively safe and non-toxic to rats (Lorke, 1983) in acute usage. These results are comparable to those of Tan *et al.* (2007) who obtained an LD_50_ greater than 5000 mg/kg with aqueous extract of *E. chlorantha* stem bark in rats by oral route. Agbaje and Onabanjo (1994) had observed also itching; leading to body scratching that lasted about 10 minutes in mice without any fatality and a LD_50_ of 7000 mg/kg and 43.65 g/kg with the ethanolic and aqueous extract, respectively, of *E. chlorantha* stem bark via oral route. Consequently, this suggests that the extract had a moderately high safety margin.

It was also observed that the plant extract caused a slight but insignificant decrease in the body weight of the test animals; this may be due to a decrease in appetite, which may be secondary to a feeling of fullness after administration of the extract. It may also be due to the effect of the plant on the body fat metabolism. This, however, remained to be rationalized. Tan *et al.* (2007) had reported an increase in body weight in an acute toxicity study of the aqueous stem bark extract of the plant.

With accurate determination of haematologic parameters, about 80% of haematologic diagnoses can be made and information collected to evaluate the stage of a particular disease or to diagnose some diseases that may not be directly related to the haematopoietic system (Zorriehzahra *et al.,* 2010). The significant decrease in the levels of PCV, haemoglobin concentration and red blood cell counts with increasing doses showed that the stem bark of this plant could cause anaemia in animals that browse on them. The anaemia may however be normocytic normochromic Acute loss of blood cell mass as seen in haemorrhage and haemolysis is the major cause of normochromic normocytic anaemia (Ford, 2013). The observed decrease in RBC counts in *E. chlorantha* treated groups may have been due to haemolysis mediated via the phytochemical components of the extract, or the extract may have caused failure of erythropoietin production (Chattopadhyay, 2003). In this study, *E. chlorantha* was attributed to contain saponins, which are known to have deleterious haemolysing effect on circulating erythrocytes (Sofowora, 1993). This is in consonance with the findings of Igwe and Onabanjo (1989) who reported that *Annona senegalensis*, which also belongs to the Annonaceae family, may induce anaemia in rats via its constituents.

The observed significant increase (*p<*0.05) in values of WBC at 2000 and 3000 mg/kg may be attributed to stimulation of the immune system that might have been caused by chemical and secondary infections. Information generated from white blood cell count, without differential count may only be partial and in some cases misleading. Thus, granulocytes are estimated in differential count to establish the nature of infection. The results of the present study suggested that differential counts were not significantly different in the control and test groups; this may be indicative of the non-allergic nature of the extracts. This may also indicate that the extract did not compromise the immune status of the animals. Our findings agree with Adebayo *et al.* (2010) who reported that toxic plants do not produce direct effect on WBC and its differentials but contrasted with Aboyade *et al.* (2009) who reported decrease in WBC following administration of *Solanum aculeastrum* to Wistar rats. *E. chlorantha* extract exerted a significant reduction in the platelets, indicating thrombocytopenia. Thrombocytopenia is indicative of an impairment of haemostatic function and herbal remedies are cited among the causes of drug-induced thrombocytopenia (Cheesbrough, 2000). Platelet aggregation plays a pivotal role in the physiopathology of thrombotic diseases. In addition, platelet activity may play a major role in the development as well as in the stability of atherosclerotic plaques and as a consequence, anti-platelet agents have been used clinically in patients at risk for myocardial ischaemia, unstable angina and acute myocardial infarction (Gorge, 2000; Kang, 2001). The extract therefore might be useful in the management of cardiovascular diseases. Tan *et al*. (2007) had earlier reported thrombocytopenia in the sub-acute toxicity study of the aqueous extract of the plant. The significant decrease in haematologic parameters due to *Enantia chlorantha* in a dose-dependent manner may be an indication of toxicity in high doses. Moody *et al.* (2007) had earlier reported the same findings.

The changes in mean value of total proteins were not statistically significant, suggesting that the extract at these high doses does not affect the protein synthesis function of the liver. Serum from animals treated with *E. chlorantha* did not show any significant increase in ALT, AST, ALP activity levels, indicating that the plant extract produced no significant change in the activities of serum enzymes.

Though the administration of the extract at different dose levels to rats did not result in any significant change in the level of liver function markers, such as aspartate amino transferase (AST), alanine amino transferase (ALT), total bilirubin, alkaline phosphatase, albumin, as compared with the controls. Inflammatory changes seen histologically (characterised by infiltration of lymphocyte at portal and central veins of the liver) at 2000 and 3000 mg/kg body weight respectively showed that the extract at these doses exerted a deleterious effect on the liver. The liver is capable of regenerating damaged tissue, hence liver function may not be impaired early following an insult from a toxicant. Again, liver congestion could be attributed, in part, to its role in biotransformation of xenobiotics. Histological changes in the heart and kidney of the test groups were mainly mild to marked congestion of coronary and renal tissues, respectively, at doses above 1000 mg/kg. The kidney is an excretory organ that removes metabolised and non-metabolised toxic materials from the body; it would thus be exposed to high concentrations of noxious materials that could cause lesions. We hereby propose that the extract did cause treatment related disorder in kidney structure. Histopathological examination indicated possible acute cardiotoxicity and nephrotoxicity.

The observed lesions in the kidneys and heart of rats given 1000 mg mg/kg and above showed that the extract exerted multiple organ toxicities at these doses; it should therefore be used with caution in patients with known history of liver, kidney and heart related disorders.

## Conclusion

The therapeutic application of the extract of *E. chlorantha* appears to be quite safe at doses below 1000 mg/kg body weight, as found in this study. It is suggested that reproductive toxicological studies of the extract should be carried out. The obtained results suggest that oral application of *Enanthia chlorantha* may not produce severe toxic effects at doses lower than 500mg extract/kg body weight. Given the wide ethnopharmacological applications of *E. chlorantha*, the present toxicity results constitute safety information that can be used in obtaining regulatory approval for its commercialisation.
